# Lightweight bobbin yarn detection model for auto-coner with yarn bank

**DOI:** 10.1038/s41598-024-67196-2

**Published:** 2024-07-12

**Authors:** Ning Dai, Xiaohan Hu, Kaixin Xu, Xudong Hu, Yanhong Yuan, Jiajia Tu

**Affiliations:** 1 Key Laboratory of Modern Textile Machinery and Technology of Zhejiang Province, Zhejiang Sci-Tech University, Hangzhou, 310018 China; 2 School of Automation, Zhejiang Institute of Mechanical & Electrical Engineering, Hangzhou, 310053 China

**Keywords:** Bobbin yarn defect, YOLOv8, LSKUnit, Biformer, FasterNeck, Engineering, Electrical and electronic engineering, Mechanical engineering

## Abstract

The automated replacement of empty tubes in the yarn bank is a critical step in the process of automatic winding machines with yarn banks, as the real-time detection of depleted yarn on spools and accurate positioning of empty tubes directly impact the production efficiency of winding machines. Addressing the shortcomings of traditional methods, such as poor adaptability and low sensitivity in optical and visual tube detection, and aiming to reduce the computational and detection time costs introduced by neural networks, this paper proposes a lightweight yarn spool detection model based on YOLOv8. The model utilizes Darknet-53 as the backbone network, and due to the dense spatial distribution of yarn spool targets, it incorporates large selective kernel units to enhance the recognition and positioning of dense targets. To address the issue of excessive focus on local features by convolutional neural networks, a bi-level routing attention mechanism is introduced to capture long-distance dependencies dynamically. Furthermore, to balance accuracy and detection speed, a FasterNeck is constructed as the neck network, replacing the original convolutional blocks with Ghost convolutions and integrating with FasterNet. This design minimizes the sacrifice of detection accuracy while achieving a significant improvement in inference speed. Lastly, the model employs weighted IoU with a dynamic focusing mechanism as the bounding box loss function. Experimental results on a custom yarn spool dataset demonstrate a notable improvement over the baseline model, with a high-confidence mAP of 94.2% and a compact weight size of only 4.9 MB. The detection speed reaches 223FPS, meeting the requirements for industrial deployment and real-time detection.

## Introduction

For the textile industry, the comprehensive transition into smart factories is an inevitable trend^1^, and automatic winding machines are widely applied in the practical production of textile factories^2^. Achieving the automation of the production process of automatic winding machines and reducing manual intervention necessitate yarn spool recognition as an essential task. In recent years, the renovation projects of automatic yarn spool changing technology in yarn bank-style automatic winding machines have garnered considerable attention from various sectors within the industry^3^. Therefore, in the context of the era of smart manufacturing, addressing the challenge of enhancing the stability, accuracy, and real-time performance of yarn spool recognition has become an urgent priority for numerous contemporary textile enterprises^4^.

Traditional yarn spool detection techniques primarily rely on photocell sensors and traditional computer vision algorithms^5^. Yang et al.^6^ utilized traditional vision methods for image preprocessing, employing support vector machines to recognize yarn spools based on hue and saturation information in the images. Zhang et al.^7^ applied HSV color space transformation, performing preprocessing such as color deviation correction and filtering on yarn spool images, and ultimately used template matching for recognition. Gao et al.^8^ enhanced target texture information using Gabor filters, followed by texture feature construction through main color extraction and color difference calculation in each partition. They achieved yarn spool recognition with a multi-class SVM. These traditional visual detection algorithms often require significant manual intervention, and tasks such as feature extraction and preprocessing heavily rely on human expertise. This results in limited generalization ability and high manual labor costs^9^. Additionally, the intricate detection steps lead to drawbacks such as slow detection speeds^10^, making them less suitable for the environment of textile factories.

Due to the limitations of traditional visual detection algorithms, researchers have gradually shifted their focus to neural network-based detection algorithms. In recent years, with the development of deep learning technology, deep learning-based object detection algorithms are gradually replacing traditional ones in various industrial fields due to their strong generalization ability^11^, high accuracy^12^, and robustness. Currently applied algorithms for industrial object detection include CNN^13^, GAN^14^, and ViT^15^, among which CNN has the earliest development and the widest application in industrial production^16^. CNNs used for object detection are mainly divided into two categories: single-stage detection algorithms represented by YOLO^17^ and SSD^18^, and two-stage detection algorithms represented by RCNN^19^. Two-stage detection algorithms require the delineation of candidate regions, followed by feature extraction of target features through convolution, and finally, SVM classification. Subsequently, Girshick^20^, in conjunction with SPPNet^21^, proposed a faster and more robust Fast R-CNN, using VGG16^22^ as the Backbone while achieving end-to-end training. Ren et al.^23^ further proposed the Faster R-CNN model based on this, utilizing a Region Proposal Network (RPN) and an Anchor mechanism to integrate region generation with the convolution network, achieving a breakthrough in detection speed. While two-stage detection algorithms have certain advantages in detection accuracy^24^, their detection speed is challenging to meet the requirements of real-time industrial detection. The YOLO series, after years of development, has evolved to YOLOv8. Its model features fast detection speed, high accuracy, and high portability and portability, making it well-suited for industrial detection environments. The Backbone of YOLOv8 combines the design principles of CSPDarkNet53^25^ and YOLOv7^26^, using the C2f module instead of the C3 module in YOLOv5, providing the model with a richer gradient flow. Varghese et al.^27^ evaluated the performance of YOLOv8 compared to other YOLO series detection models on the COCO dataset, focusing on Average Precision Across Scales (APAS) and Frames Per Second (FPS). The results indicate that YOLOv8 achieved the highest values in both metrics. Specifically, YOLOv8's APAS was 2.4% higher than YOLOv7, and its detection frame rate was 10 FPS higher than YOLOv5. These data demonstrate the superior performance of YOLOv8 in target detection tasks.

**Figure 1:**
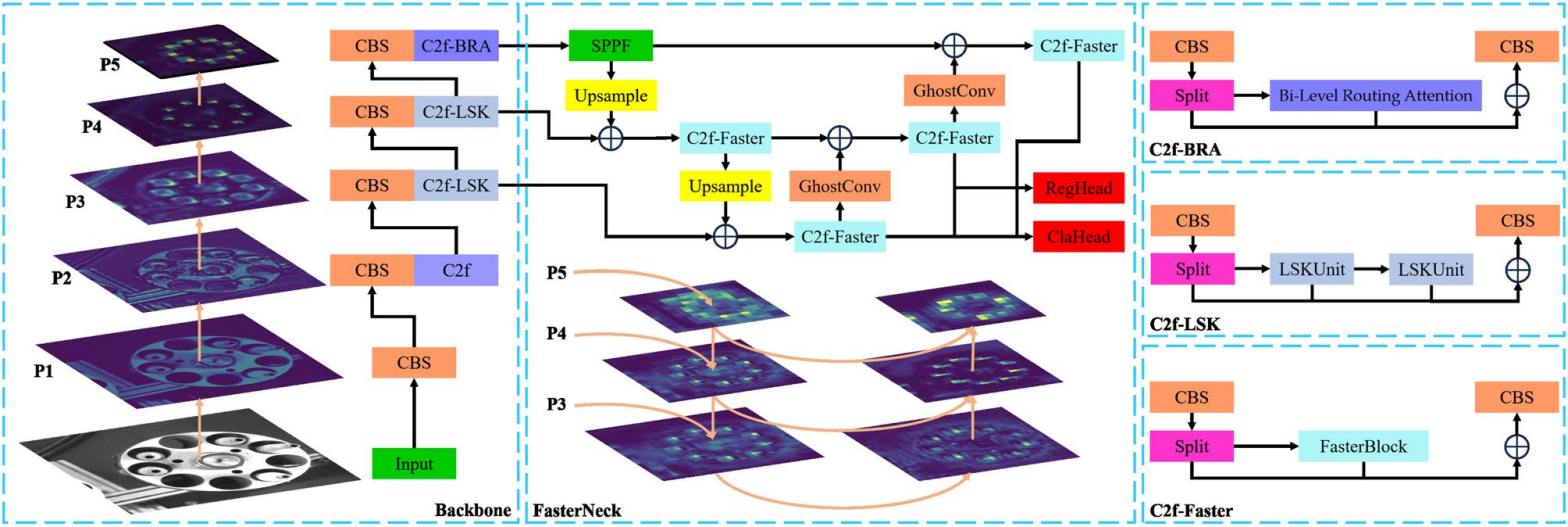
The improved model architecture diagram. denotes the concatenation operation.

However, the improvement in detection accuracy often comes with a significant increase in computational costs, time costs, and high deployment expenses, making it cost-prohibitive for most textile enterprises. To address these challenges, this paper proposes a lightweight, efficient, and high-precision yarn tube detection algorithm based on YOLOv8. The model, as illustrated in Fig. 1, achieves higher detection accuracy with a lightweight design, faster detection speed, and is poised to support real-time detection in industrial applications. The primary contributions of this paper are as follows:

1. Improved the model's Backbone by incorporating LSKUnit^28^ and C2f modules. By leveraging the multi-gradient flow characteristics of the C2f module along with LSKUnit's large kernel selection mechanism, the model enhances the detection performance for densely distributed targets, strengthening the feature extraction capabilities of the Backbone.
2. Utilized the dynamic sparse attention mechanism of Biformer^29^, known as BRA (Bi-Level Routing Attention), to enhance the model's ability to capture global information. This compensates for convolutional networks overly focusing on local features at the expense of global features, as demonstrated in experiments that show improved performance on the yarn tube dataset.
3. Replaced the original convolutional modules and C2f modules with lightweight GhostConv^30^ modules and C2f-Faster modules, proposing a lightweight feature pyramid structure called FasterNeck. This significant reduction in model parameters and volume resulted in a noticeable increase in detection speed.
4. Introduced WIoU^31^ as the loss function for bounding box regression. In comparison to the baseline network's CIoU loss function, WIoU strengthens the model's focus on medium-quality training data, reduces the weight of low-quality training data, thereby enhancing the model's generalization ability and robustness.
5. Finally, conducted ablation experiments using a custom dataset to validate the improved model's performance. The experimental results demonstrate enhanced yarn tube recognition capabilities, faster detection speed, and a smaller model volume, reducing deployment costs and meeting the requirements of the textile industry.

## Materials and methods

### Large selective kernel unit

**Figure 2:**
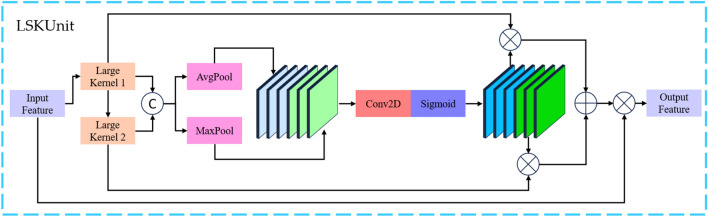
LSKUnit attention module structure diagram. In the diagram, $\oplus$ represents element-wise addition, $\otimes$ denotes element-wise multiplication, and © signifies channel-wise concatenation operation.

LSKNet (large selective kernel net), proposed by Yuxuan Li and colleagues, is designed to address challenges in the field of sensor image detection. The model achieves focus on the background regions most relevant to the target under examination by decomposing and selecting large convolutional kernels. The LSKUnit Attention module structure is shown in Fig. 2.

The LSKUnit serves as the foundational module of LSKNet, and its structure is illustrated in the above diagram. The input features of the module undergo convolution with multiple decomposed large convolutional kernels, yielding feature maps corresponding to different large receptive fields. Subsequently, these feature maps are concatenated along the channel dimension. Following this concatenation, average pooling and max pooling layers are applied to effectively extract spatial feature information. The mathematical expression for this feature operation is as follows:

1 $$ F_{1} = Kernel\;1(F_{in} );F_{2} = Kernel\;2(F_{in} )F $$

2 $$ F_{k} = [F_{1} ;F_{2} ] $$

3 $$ F_{pool} = [AvgPool(F_{k} );MaxPool(F_{k} )] $$

In the above equation, $Kernel\;1(F_{in} )$ refers to the convolution of the input feature $F$ with the first convolutional kernel. $AvgPool$ and $MaxPool$ represent average pooling and max pooling, respectively.

After obtaining spatial features, a convolutional layer followed by a Sigmoid activation function is employed to facilitate the fusion and interaction of different spatial features. This process results in the generation of attention module weights. Subsequently, these weights are used to weight the features outputted by the large convolutional kernels, producing the final output features of the module. The mathematical expression for this feature operation is as follows:

4 $$ F_{attn1;attn2} = Sigmoid(Conv(F_{pool} )) $$

5 $$ F_{out} = (F_{attn1} *F_{1} + F_{attn2} *F_{2} )*F_{in} $$

In the above equation, $Conv$ represents the Conv2D module, and $Sigmoid$ denotes the sigmoid activation function.

**Figure 3:**
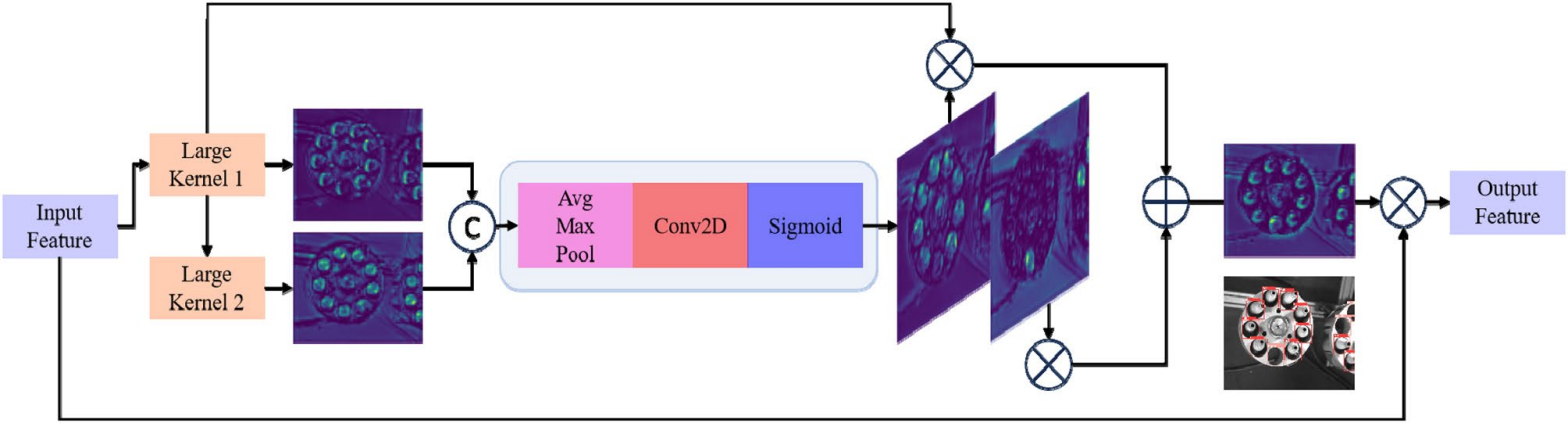
Schematic diagram of LSKUnit. In the diagram, $\oplus$ represents element-wise addition, $\otimes$ denotes element-wise multiplication, and © signifies channel-wise concatenation operation.

By incorporating the adaptive kernel adjustment function of the LSKUnit, the features input into the LSKUnit undergo a serial operation of two large convolutional kernels, achieving dynamic receptive field adjustment. This design enables the LSKUnit to adaptively extract different scale features of the bobbin. Compared to using a single larger convolutional kernel, the serial approach with multiple large kernels offers advantages in both computational speed and parameter efficiency. Additionally, the use of multiple convolutional kernels of different sizes allows the module to generate spatial features with multi-scale and multi-receptive field characteristics, enriching the target features and enhancing the backbone network's ability to focus on background features at a lower computational cost. The schematic diagram of LSKUnit is shown in Fig. 3.

### Biformer

As is well-known, compared to convolutional neural networks, the core advantage of Transformers lies in their ability to capture long-range dependencies through self-attention mechanisms. While this structure can enhance model performance to a certain extent, it comes with the drawback that traditional self-attention modules consume a significant amount of memory and entail high computational costs.

**Figure 4:**
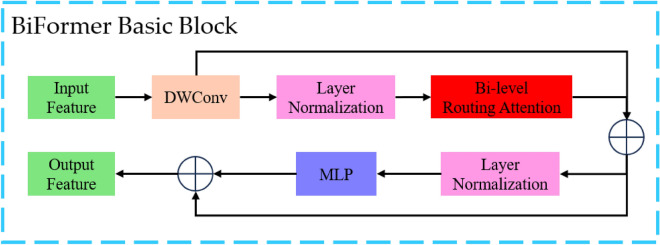
Biformer module structure diagram. In the diagram, denotes concatenation operation, and DWConv represents the depthwise separable convolution module.

To address this issue, Lei Zhu et al.^32^ proposed the Biformer model. This model reduces computational costs by introducing a two-level dynamic sparse attention mechanism, known as BRA. The key idea is to initially apply coarse-grained filtering to the input features, ignoring most of the irrelevant key-value pairs, and then perform fine-grained token-to-token attention on the remaining regions. The module structure diagram is shown in Fig. 4.

The Biformer module follows a similar architecture to many Transformer models, consisting of a self-attention unit and a multilayer perceptron unit. Unlike other ViT models, Biformer introduces a novel self-attention unit called BRA (Bi-level Routing Attention). In the BRA module, the input features with a height of H, width of W, and C channels are divided into S*S regions. Each region is then processed with learnable weight matrices $W^{q}$, $W^{k}$, and $W^{v}$ to obtain three vectors: Q (queries), K (keys), and V (values). The calculation formula is as follows:

6 $$ F_{in} \in R^{H \times W \times C} \to X \in R^{{S^{2} \times \frac{HW}{{S^{2} }} \times C}} $$

7 $$ Q = XW^{q} ;K = XW^{k} ;V = XW^{v} $$

The calculation formula for the self-attention mechanism is as follows:

8 $$ Attention(Q,K,V) = Soft\max (\frac{{QK^{T} }}{\sqrt C })V $$

The constant $C$ in the equation is introduced for gradient stability normalization. From the above formula, it can be observed that the self-attention mechanism involves a weighted operation on the V matrix. The weight values $QK^{T}$ are used to measure the correlation of features from other different regions with the selected region Q. A top-k algorithm is applied to select the k regions that exhibit the highest correlation with the current selected region Q, achieving a coarse-grained key-value filtering.

Subsequently, the obtained key-value pairs of the k most relevant regions to the currently selected region are merged, and fine-grained token-to-token attention calculation is performed:

9 $$ I_{n} = index(topk(QK^{T} )),n \in 1,2, \cdot \cdot \cdot ,k $$

10 $$ K_{g} = Gather(K,I_{n} );V_{g} = Gather(V,I_{n} ) $$

11 $$ Attention(Q,K_{g} ,V_{g} ) = Soft\max \left( {\frac{{QK_{g}^{T} }}{\sqrt C }} \right)V_{g} $$

**Figure 5:**
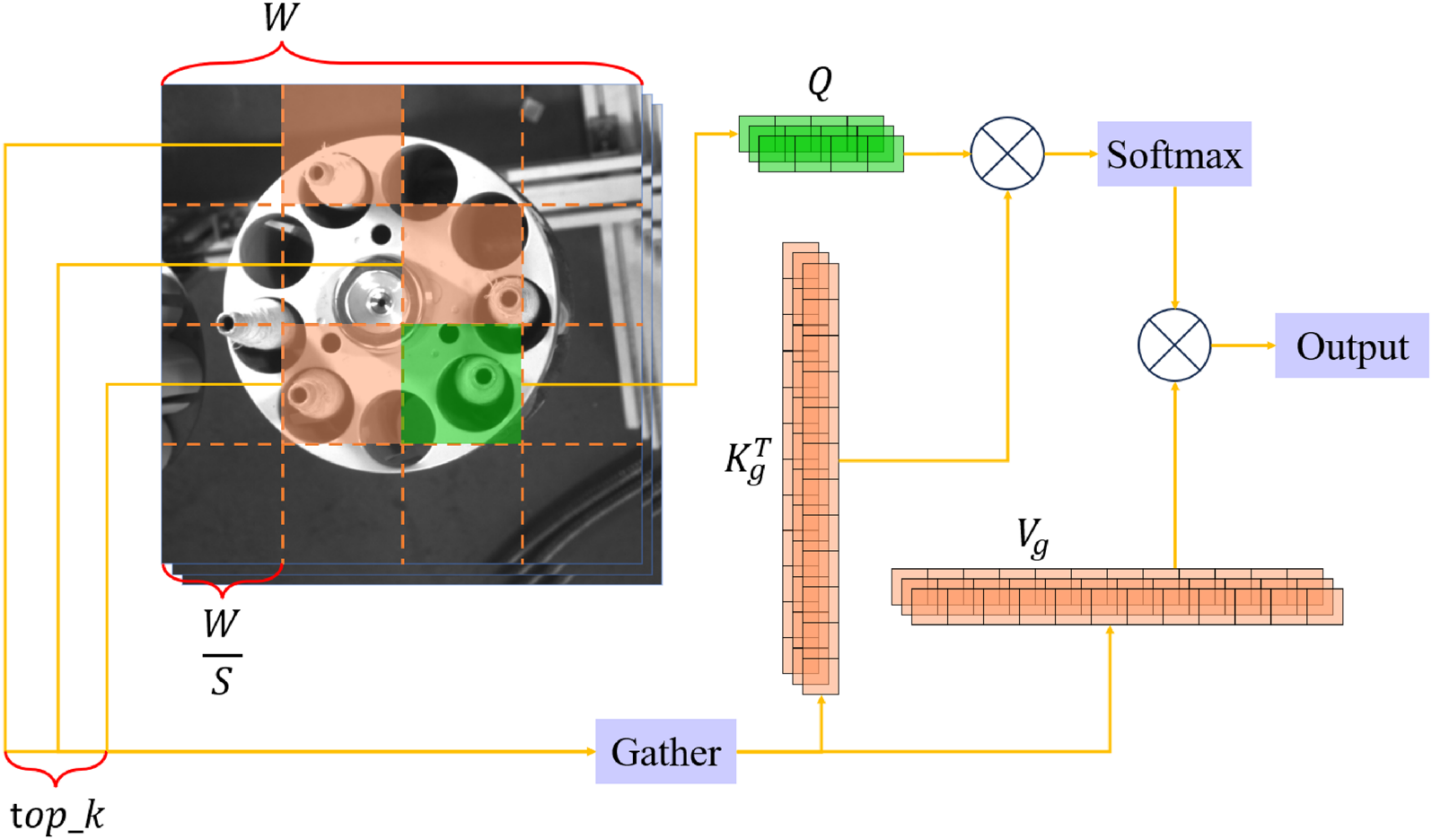
Bi-level routing attention principle schematic diagram. In the diagram, represents matrix multiplication.

By introducing Biformer to enhance the model's ability to capture long-distance dependencies, the network's awareness of contextual features is strengthened. Additionally, due to the reduced computational complexity and faster attention operations of Bi-level Routing Attention compared to other self-attention mechanisms, a better balance is achieved between model accuracy and detection speed. The schematic diagram of Bi-level Routing Attention is shown in Fig. 5.

### Lightweight improvement of neck

The advancement of deep neural networks has propelled significant progress in various computer vision tasks, leading to higher accuracy in areas such as object detection and image recognition. However, this progress is accompanied by larger models, deeper network structures, and increased computational costs. For less complex downstream computer vision tasks, achieving a balance between accuracy and speed is crucial to effectively apply the technology in practical scenarios.

**Figure 6:**
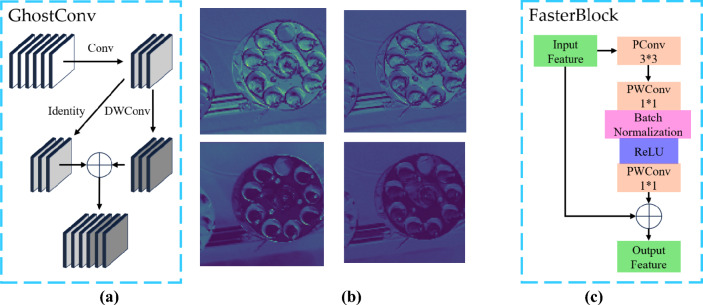
(**a**) GhostConv principle schematic diagram; (**b**) Example of a few Ghost pairs; (**c**) FasterBlock structure diagram.

GhostConv, introduced by Han^9^ and others, was developed based on the observation that the feature maps extracted by the backbone network of ResNet-50 often include many highly similar feature maps, referred to as Ghost pairs. The researchers found a positive correlation between these Ghost pairs and the model's feature extraction capability. Therefore, they proposed using a simple linear transformation to generate multiple sets of Ghost pairs, aiming to enhance the model's feature extraction ability while reducing the computational cost associated with convolutions. This is the core idea behind GhostNet. In the construction of the module, the implementation of the simple linear transformation utilizes DWConv (Depthwise Convolution). An example of GhostConv schematically paired with a small amount of Ghost is shown in Fig. 6a,b.

FasterBlock was introduced by Jierun Chen and collaborators in response to the limitations of classic lightweight convolutions such as DWConv (Depthwise Convolution), which primarily focus on reducing FLOPs (Floating-Point Operations) without effectively optimizing FLOPS (Floating-Point Operations per Second). Experimental findings revealed that frequent memory access in DWConv resulted in a lack of substantial speed improvement in actual computational processing units (CPU or GPU) during correlation operations.To address this issue, Jierun Chen and the team developed a technique called PConv (Partial Convolution) and used it as the foundation to construct FasterNet. FasterNet incorporates a significant number of PConv operations, which are a form of partial convolution, meaning that only a subset of channels in the input feature is convolved. This approach significantly reduces both computational and memory access demands compared to full convolutions. However, this convolution method may neglect information from certain input feature channels. To mitigate this, a point-wise convolution (Point-Wise Convolution) is applied after PConv, enhancing the utilization of other channels in the input feature without incurring excessive computational costs. The FasterBlock structure is shown in Fig. 6c.

### Loss function improvement

In YOLOv8, the CIoU (Complete Intersection over Union) is employed as the bounding box regression loss function. CIoU takes into account factors such as the overlap area, center distance, and aspect ratio between the predicted bounding box and the ground truth box. It achieves better bounding box regression performance compared to the IoU loss function. However, datasets collected in industrial applications often vary in data quality. CIoU cannot adapt its loss penalty based on the quality of different datasets. This limitation can cause the model to overly focus on either challenging or easily detectable targets, leading to a decrease in the model's robustness.

To address this issue, this paper utilizes WIoU (Weighted IoU) as the bounding box regression loss function. WIoU incorporates a non-monotonic dynamic focusing mechanism, and its specific implementation is as follows:

12 $$ \beta = \frac{{loss_{IoU} }}{{\overline{{loss_{IoU} }} }} \in [0, + \infty );\gamma = \frac{\beta }{{\delta \alpha^{\beta - \delta } }} $$

In the above equation, $\beta$ is defined as the outlying degree of the current data. A larger value indicates lower data quality, making it more challenging to detect, while a smaller value suggests higher quality, making it easier to detect. $\gamma$ is a non-monotonic dynamic focusing coefficient, and $\delta$ and $\alpha$ are hyperparameters. When $\beta$ equals $\delta$, $\gamma$ is set to 1, indicating no weighting of the loss for training data. Since $\overline{{loss_{IoU} }}$ represents the average IoU loss of the training-involved data, which can be self-learned, $\gamma$ dynamically changes with the progress of training. The calculation formula for WIoU is as follows:

13 $$ loss_{WIoU} = \gamma *e^{{[\frac{{(x - x_{gt} )^{2} + (y - y_{gt} )^{2} }}{{W_{g}^{2} + H_{g}^{2} }}]}} *loss_{IoU} $$

$(x,y)$ represents the center position of the ground truth bounding box, and $(x_{gt} ,y_{gt} )$ represents the center position of the predicted bounding box. The equation above indicates that this loss function is composed of a non-monotonic dynamic focusing coefficient, a center distance penalty mechanism, and the original IoU loss function.

Compared to the original CIoU in the model, WIoU can dynamically adjust the loss weights for different detection difficulty during training. This enables the model to focus more on moderately challenging training data, thereby improving the model's robustness while reducing the risk of overfitting.

## Experiment and analysis

### Experimental platform and dataset

**Figure 7:**
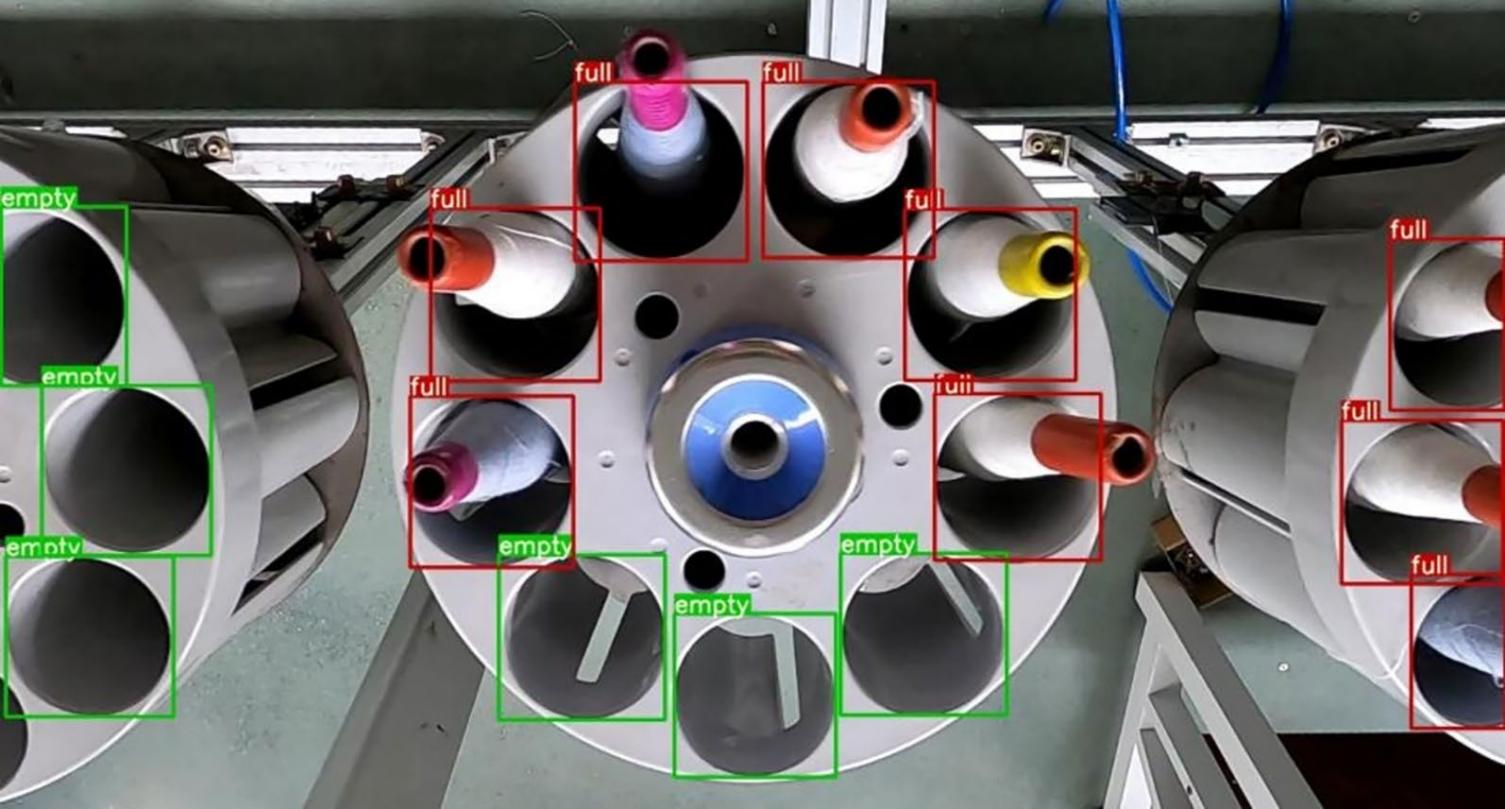
Annotation example of the self-made bobbin dataset. Bobbins inside the bobbin rack are annotated as "full," and those without bobbins are annotated as "empty."

The software and hardware information of the experimental platform is shown in Table 1. The dataset used in the experiments comprises two parts: one is collected from idle yarn storage in a textile factory's production line, and the other consists of directly captured images of yarn tubes and yarn storage. After basic image augmentations such as horizontal flipping and vertical flipping, a total of 628 images were obtained. The dataset contains approximately 12,000 annotated targets, classified into categories of with yarn tubes and without yarn tubes. The dataset was partitioned into training, validation, and test sets in a ratio of 8:1:1. The labeling of the target is shown in Fig. 7 below:

**Table 1:** Hardware and software information of the experimental platform.

Items	Version and parameters
Operating system _train	Windows 10 Pro 22H2 Intel(R) Xeon(R) Gold 6248R
CPU_train	
GPU_train	NVIDIA RTX A6000 64 GB
RAM_train	DDR4 2933 MHz 128 GB
Operating system _test	Windows 11 Home 22H2
CPU_test	Intel(R) Core(TM) i9-12900H
GPU_test	NVIDIA RTX 3060 Laptop GPU
RAM_test	DDR5 4800 MHz 16 GB
Pytorch version	Python-3.9 Torch-1.12.0 Cuda-11.6.0

To ensure the effectiveness and rigor of the experimental result analysis, the study employed precision (P), recall (R), and mean average precision (mAP) as the primary evaluation metrics for the models. In the comparison of models with different structures, model size and inference frames were used as additional reference metrics.

14 $$ P = \frac{TP}{{TP + FP}},R = \frac{TP}{{TP + FN}} $$

15 $$ AC = \frac{TP + TN}{{TP + FP + TN + FN}},mAP = \frac{{\sum {AC} }}{C} $$

where TP is the number of true positive samples detected, FP is the number of false positive samples (misclassified as negative), TN is the number of true negative samples detected as negative, FN is the number of false negative samples (missed positive samples), $\sum {AC}$ is the overall accuracy across all classes, and C is the number of defect categories.

### Experiment on network structure improvement

Given the considerations of industrial application costs and the difficulty of the empty bobbin visual detection task, this paper utilized the YOLOv8 nano baseline model as the benchmark for comparative experiments in the network structure improvement. Compared to other YOLOv8 baseline models, the nano model has the advantages of small size, fast inference speed, and low computational cost. It is one of the most suitable models for deployment in industrial real-time detection. However, due to its minimal network depth and width, its accuracy is not as high as the other YOLOv8 baseline models. The performance of YOLOv8n on the bobbin dataset is shown in the Table 2 below:

**Table 2:** Performance of the baseline model YOLOv8n on the dataset used in this study.

Class	P	R	mAP@0.5	mAP@0.5–0.95	WeightSize	Parameters	Speed
ALL	99.4%	99.7%	99.5%	93.4%	6.2 MB	3 M	177fps
Full	1	99.7%	99.5%	94.7%			
Empty	98.9%	99.8%	99.5%	92.1%			

^1^In this paper, unless otherwise specified, mAP@0.5 refers to the model's average precision at a confidence threshold of 0.5, and mAP@0.5–0.95 refers to the average precision across different confidence thresholds ranging from 0.5 to 0.95 with a step size of 0.05.

From the above experimental results, it can be observed that the model achieves a high level of average accuracy at a confidence threshold of 0.5. However, as the confidence threshold increases, the precision significantly decreases, indicating the occurrence of false positives or false negatives within the high-confidence interval. Additionally, there is substantial room for improvement in the model's detection speed.

To demonstrate the performance improvement of the model on the bobbin dataset with the introduction of LSKBlock and Biformer, experiments were conducted on the YOLOv8n model. The experimental results are shown in Table 3 below:

**Table 3:** Performance improvement experiment results.

No.	Model	P	R	mAP@0.5	mAP@0.5–0.95	WeightSize	Parameters	Speed
0	Baseline	99.4%	99.7%	99.5%	93.4%	6.2 MB	3 M	177fps
1	+LSKUnit	99.4%	99.8%	99.5%	93.8% (↑0.4%)	6.5 MB	3.12 M	238fps
2	+Biformer	99.8%	99.6%	99.5%	93.5% (↑0.1%)	6.5 MB	3.15 M	222fps
3	+C2f.-LSK	99.4%	99.7%	99.5%	93.8% (↑0.4%)	6.2 MB	2.98 M	254fps
4	+C2f.-BRA	99.1%	99.8%	99.5%	93.9% (↑0.5%)	6.1 MB	2.92 M	234fps
5	+C2f.-LSK +C2f.-BRA	99.7%	99.7%	99.5%	93.8%(↑0.4%)	6.1 MB	2.90 M	230fps

From the Table 3 above, it can be observed that the LSK attention mechanism and Biformer perform well on the dataset used in this study. The data from Experiment Groups 1 and 2 indicate that the introduction of LSKUnit improves the model's average accuracy by 0.4%, and the Biformer module further enhances the average accuracy by 0.1%.

When combining the above modules with the C2f module, replacing the original Bottleneck module with the new attention mechanism module, and allowing the model to receive attention-weighted multi-gradient information, the model achieves local feature fusion with minimal computational cost. This enhances the accuracy of the model while maintaining its lightweight nature. Experiment data from Groups 3 and 4 show that, with the introduction of the C2f-LSK module and C2f-BRA module, the baseline model's mAP is improved by 0.4% and 0.5%, respectively, with varying degrees of improvement in detection speed. In Experiment 5, the C2f-LSK module and the C2f-BRA module were combined and applied to the backbone of the baseline model. The C2f-LSK module, through its mechanism of dynamically adjusting the receptive field, significantly improved the model's performance in detecting dense targets, enhancing both detection accuracy and speed. The C2f-BRA module, by enhancing the model's ability to capture global information and integrate contextual features, further enriched the target feature representation, improving the model's performance in complex backgrounds. Experimental data show that the average accuracy of the combined model improved by 0.4%, and the detection speed increased to 230 fps.

**Figure 8:**
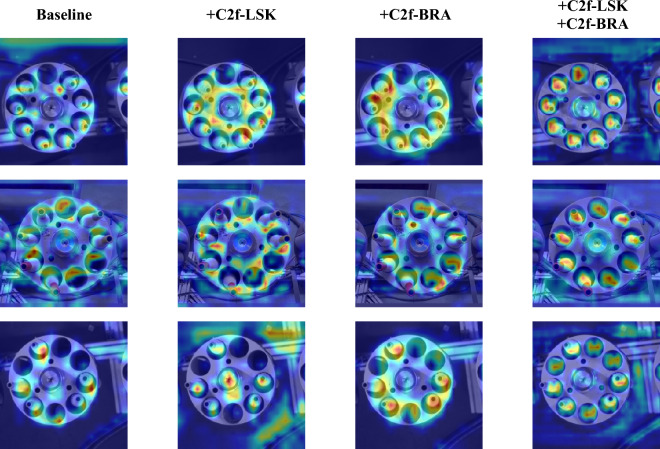
Visualization comparison of heatmap data under the influence of different attention mechanisms.

The heatmaps in Fig. 8 illustrate the impact of different attention mechanisms on feature extraction. The colors in the heatmaps indicate the model's attention to different regions, with red areas representing regions of higher attention and blue areas representing regions of lower attention. From the heatmaps, it can be seen that the baseline model (Baseline) focuses primarily on target regions but has some omissions in feature extraction within complex backgrounds, affecting detection accuracy at high confidence levels. In contrast, the C2f-LSK module's heatmap shows more uniform attention across regions, particularly with more detailed feature extraction at the edges of target objects, enhancing the model's performance in complex backgrounds. The C2f-BRA module increases attention to target regions, enriches feature extraction details, and further improves background feature extraction. The combined C2f-LSK and C2f-BRA module leverages the strengths of both, exhibiting multi-scale and multi-receptive field characteristics in the heatmaps, making target features more prominent and background feature extraction more comprehensive. This combination leads to an overall performance improvement. These experimental results strongly validate the effectiveness of the proposed structure. The experiments demonstrate that the improved C2f-LSK and C2f-BRA modules enhance the model's average accuracy on the bobbin dataset without compromising detection speed..

Therefore, this paper selects the improved C2f-LSK module to enhance the model's feature perception capability for dense yarns. Through its multi-scale large kernel selection mechanism, the module enables the model to focus on both the foreground and background of the detected target, reducing false positives and false negatives. Simultaneously, the introduction of the C2f-BRA module prevents the model from overly focusing on local information and strengthens the model's perception of global information.

In addition, for the textile industry, achieving a good balance between detection efficiency, accuracy, robustness, and lightweight characteristics is crucial. If any one of these factors is limited, it becomes challenging to apply the model to actual industrial production. Therefore, to balance production costs and detection performance, this paper conducts lightweight improvement experiments on the model, and the results are shown in the Table 4:

**Table 4:** Lightweight improvement experimental results.

No	Model	P	R	mAP@0.5	mAP@0.5–0.95	WeightSize	Parameters	Speed
0	Baseline	99.4%	99.7%	99.5%	93.4%	6.2 MB	3 M	177fps
1	+DBBNet	1	99.5%	99.5%	93.4%	6.9 MB	3.21 M	213fps
2	+GhostNet	99.5%	99.5%	99.5%	93.2% (↓0.2%)	4.0 (↓2.2)MB	1.86 (↓1.14)M	256 (↑79)fps
3	+FasterNet	99.3%	99.9%	99.5%	92.3%	9.8 MB	4.83 M	196fps
4	+SlimNeck	99.5%	99.5%	99.5%	93.5%	5.9 MB	2.81 M	232fps
5	+GhostConv	99.5%	99.7%	99.5%	93.9% (↑0.5%)	6.1 MB	2.91 M	263 (↑86)fps
6	+C2f.-Faster	99.7%	99.4%	99.5%	93.7% (↑0.3%)	5.2 MB	2.49 M	276 (↑99)fps

The results of the above experiments, specifically the 5th group, demonstrate that replacing the ordinary convolutions in the model's Neck part with GhostConv leads to a 0.5% improvement in accuracy, and the detection speed increases by 86FPS. Experiment 6 shows that using the C2f-Faster module to replace the C2f module in the baseline model's Neck significantly improves the detection speed, and the average accuracy of the model also increases by 0.3%.

In summary, the proposed improved Neck structure in this paper does achieve good lightweight effects on the yarn dataset, and the model's mAP has also seen a slight increase.

Furthermore, the results of experiment group 2 indicate that replacing the Backbone of the baseline model with GhostNet reduces the model's weight size by 2.2MB, decreases the number of parameters by 1.14M, and significantly increases the detection speed. Meanwhile, the model's accuracy only experiences a minor loss of 0.2%. Considering industrial deployment costs, the improved model in this experiment group is an excellent choice as it substantially reduces model size and parameters.

In conclusion, the proposed improved model selects the C2f-LSK module and C2f-BRA module to replace the C2f module in the YOLOv8n model's Backbone, aiming to enhance the model's detection accuracy. Additionally, GhostConv and FasterBlock modules are employed to lightweight the Neck part of the baseline model, improving the model's detection speed without sacrificing accuracy.

Experiment on Loss function Improvement

The baseline model employs Complete Intersection over Union (CIoU) as the bounding box regression loss function for the network. However, in industrial applications, it is inevitable to encounter low-quality examples that include challenges like varying distances and aspect ratios. The use of geometric metrics intensifies the penalties for low-quality examples, leading to a decline in the model's generalization performance. An effective bounding box regression loss function should aim to avoid excessive focus on low-quality training data, thereby minimizing interference with the training process and enhancing the model's generalization performance.

**Figure 9:**
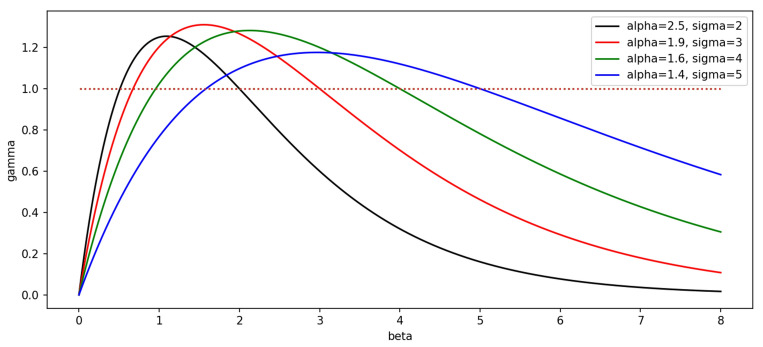
Function curve of focusing coefficient of different hyperparameter selection.

WIoU, as a bounding box regression loss function with a non-monotonic dynamic focusing property, relies on the implementation of the non-monotonic dynamic focusing mechanism, primarily governed by the focusing coefficient $\gamma$ (Eq. 12). The dynamic variation of the focusing coefficient $\gamma$ is achieved by defining the outlier degree $\beta$ as the ratio of the current anchor box's IoU loss to the average anchor box's IoU loss (Eq. 12). During training, the focusing coefficient $\gamma$ undergoes real-time iteration to achieve dynamic changes. The shape of the coefficient's function curve varies based on different hyperparameters $\delta$ and $\alpha$. The curve of the coefficient function is illustrated in the Fig. 9 below:

A smaller outlier degree, represented by $\beta$, indicates higher quality for the current bounding box, implying excellent training data. In such cases, we aim to prevent the model from overly focusing on it. Therefore, assigning a small gradient weight to it through the focusing coefficient $\gamma$ helps enhance the model's generalization capability. On the other hand, when the outlier degree $\beta$ is large, indicating poor quality for the current bounding box (low-quality training data), we assign it a very low gradient weight to avoid harmful gradients from low-quality data. For cases with moderate-quality training data, we assign a high gradient weight to it through the coefficient $\gamma$ to make the model focus on moderate-quality training data and improve overall performance.

From the figure above, it is evident that different choices of hyperparameters have a significant impact on the gradient weights assigned to training data. To identify the most suitable hyperparameters for the yarn dataset, we conducted a controlled experiment. The experimental data are shown in Table 5 below:

**Table 5:** WIoU hyperparameter selection experiment results.

Model	P	R	mAP@0.5	mAP@0.5–0.95
WIoU, *δ* = 2	99.6%	99.7%	99.5%	93.8%
WIoU, *δ* = 3	99.4%	99.9%	99.5%	94.2%
WIoU, *δ* = 4	99.8%	99.7%	99.5%	94.0%
WIoU, *δ* = 5	99.9%	99.1%	99.5%	93.7%

The experimental results of WIoU hyperparameter selection indicate that WIoU performs best on the yarn dataset when the hyperparameters are chosen as $\delta$ = 3 and $\alpha$ = 1.9. As shown in Table 6, in the subsequent comparative experiment, models utilizing WIoU achieved a 0.2% increase in average accuracy compared to models using CIoU. This confirms that, in contrast to the original CIoU, WIoU is better suited for the target detection task on the yarn dataset.

**Table 6:** The loss function improves the experimental results.

Model	P	R	mAP@0.5	mAP@0.5–0.95	WeightSize	Parameters	Speed
Ours+CIoU	99.6%	99.9%	99.5%	94.0%	4.9 MB	2.3 M	223fps
Ours+WIoU	99.4%	99.9%	99.5%	94.2% (↑0.2%)	4.9 MB	2.3 M	223fps

### Model performance experiment

To demonstrate the effectiveness of the improved network in enhancing the model's performance on the yarn dataset, this section employs an ablation study to evaluate the performance outcomes of the model enhancements. The experiment includes five variables: C2f-LSK module, C2f-BRA module, C2f-Faster module, GhostConv, and WIoU loss function. The evaluation metrics used are average precision, model weight size, parameter count, and inference speed. The results of the ablation study on the improved model proposed in this paper are presented in the Table 7 below:

**Table 7:** Results of ablation experiment.

No	C2f.-LSK	C2f.-BRA	C2f.-Faster	GhostConv	WIoU	mAP@0.5–0.95	WeightSize	Parameters	GFLOPs	Speed
0	**–**	**–**	**–**	**–**	**–**	93.4%	6.2 MB	3 M	5.2G	177fps
1	**√**	**–**	**–**	**–**	**–**	93.8% (↑0.4%)	6.2 MB	2.98 M	5.4G	254fps
2	**–**	**√**	**–**	**–**	**–**	93.9% (↑0.5%)	6.1 MB	2.92 M	5.0G	234fps
3	**√**	**√**	**–**	**–**	**–**	93.8% (↑0.4%)	6.1 MB	2.90 M	4.8G	230fps
4	**–**	**–**	**√**	**–**	**–**	93.7%	5.2 (↓1.0)MB	2.49 (↓0.5)M	4.5G	276 (↑99)fps
5	**–**	**–**	**–**	**√**	**–**	93.9%	6.1 (↓0.1)MB	2.91 (↓0.1)M	4.8G	263 (↑86)fps
6	**–**	**–**	**√**	**√**	**–**	93.9%	5.0 (↓1.2)MB	2.40 (↓0.6)M	4.2G	249 (↑72)fps
7	**√**	**√**	**√**	**√**	**–**	94.0% (↑0.6%)	4.9 (↓1.3)MB	2.30 (↓0.7)M	4.1G	223 (↑46)fps
8	**√**	**√**	**√**	**√**	**√**	94.2% (↑0.8%)	4.9 (↓1.3)MB	2.30 (↓0.7)M	4.1G	223 (↑46)fps

From the experimental data of Experiment Group 1 and Experiment Group 2, it is evident that the introduction of the improved C2f-LSK module and C2f-BRA module can effectively enhance the network's accuracy. However, the deepening of the model may exacerbate the loss of shallow features in the network. Additionally, the introduction of more convolutions also causes the model to focus more on local information. Therefore, by introducing the C2f-BRA module with a self-attention mechanism alongside the C2f-LSK module, the model's attention to global information is enhanced. The data from Experiment Group 3 shows that this improvement approach is effective, resulting in a 0.4% increase in the model's average precision.

The results from Experiment Group 4 and Experiment Group 5 demonstrate that the C2f-Faster and GhostConv modules can significantly improve the model's detection speed without causing a loss in accuracy. Experiment 6, which combines both modules, shows that the lightweight improvement in the Neck structure leads to a noticeable reduction in parameter count and weight size, along with a significant increase in detection speed.

Lastly, the data from Experiment 7 and Experiment 8 strongly confirms the superiority of the proposed structure. With the introduction of the WIoU loss function, the model's accuracy improves by 0.8% compared to the baseline model. Additionally, the detection speed increases by 46FPS, and there is a substantial reduction in model weight size and parameter count.

**Figure 10:**
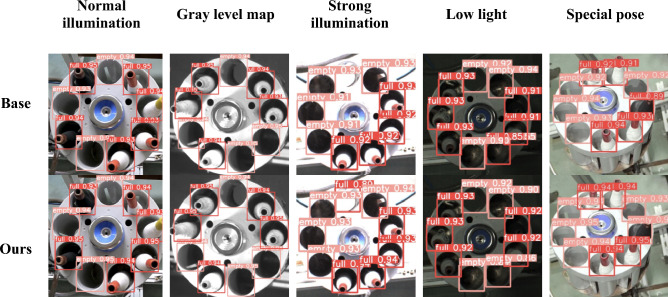
Comparison of detection effect under different conditions.

Comparison experiments of detection effects under different conditions are shown in Fig. 10. The detection results above indicate that the proposed model, achieving lightweight improvements, maintains detection performance comparable to the baseline model. Moreover, in some extreme conditions, the detection performance surpasses that of the baseline model (such as low-light environments and large pose angles, remaining on par under strong lighting). This achievement is mainly attributed to the introduction of the WIoU loss function, enhancing the model's generalization ability and enabling adaptation to various application scenarios.

To validate the performance of the improved lightweight bobbin detection network model, we compared it with current mainstream object detection algorithms. The experimental results are shown in Table 8.

**Table 8:** Different models compare experimental results.

Model	P	R	mAP@0.5–0.95	WeightSize	Parameters	GFLOPs	Speed
Faster-RCNN	93.4%	92.5%	89.9%	52.1 MB	33.6 M	153.2G	97fps
SSD	88.6%	90.3%	86.4%	9.3 MB	16.3 M	32.7G	201fps
YOLOv5n	95.8%	97.6%	91.5%	7.5 MB	5.5 M	7.9G	160fps
YOLOv8n	99.4%	99.7%	93.4%	6.2 MB	3.02 M	5.2G	177fps
Ours	99.4%	99.9%	94.2%	4.9 MB	2.30 M	4.1G	223fps

From the experimental results in Table 8, it can be seen that the Faster-RCNN network model has the highest parameter count and computational load, resulting in a larger model weight file and lower detection frame rate. Although the SSD network model has a higher detection frame rate than Faster-RCNN, its detection accuracy is lower, making it unsuitable for deployment in embedded mobile devices and industrial environments with limited computational resources. In comparison to these two-stage object detection models, the improved lightweight bobbin detection model based on YOLOv8 proposed in this paper shows superior performance in terms of detection accuracy and parameter count. Compared with the baseline model YOLOv8n, the average detection accuracy is improved by 0.8%, the parameter count is reduced by 0.72 M, and the detection frame rate is increased by 46fps, enabling effective bobbin detection in the spinning environment.

## Conclusion

This paper introduces a lightweight and high-precision small model based on an improved YOLOv8 for yarn cone detection in the yarn warehouse-type automatic winding machine, demonstrating excellent performance.

Starting from the perspective of model accuracy optimization, experiments were conducted to enhance the model's detection accuracy. The LSKUnit, featuring a large kernel selection mechanism, effectively improves the model's ability to extract shallow features. Its utilization of multiple large convolutional kernels enriches the model's output features, providing it with multiscale spatial information. Subsequently, the Biformer is employed to reinforce the model's contextual association capabilities, addressing the limited attention to global information in convolutional neural networks. Additionally, the combination of LSKUnit and Biformer with the C2f module in the baseline model is experimentally validated, highlighting the advantages of the improved C2f-LSK and C2f-BRA modules.

For industrial detection applications, with a focus on reducing model deployment costs, the model undergoes lightweight deployment improvements. FasterBlock is proven effective in accelerating both the model's detection and training processes. Consequently, integrating FasterBlock with the C2f module in the Neck section of the baseline network reduces model parameters while leveraging the C2f module's multi-gradient flow concept, allowing PWConv within FasterBlock to utilize information from different gradients, compensating for precision losses caused by PConv. Substituting GhostConv for the regular convolutional modules in the Neck section, GhostConv employs a simple linear transformation to produce convolution-like effects, reducing model parameters and computational costs. Subsequent experiments demonstrate the effectiveness of the C2f-Faster module and GhostConv in improving model detection speed and achieving model lightweighting without sacrificing accuracy.

Finally, to enhance model generalization and robustness, the WIoU loss function is employed as a replacement for CIoU. The non-monotonic dynamic focusing mechanism in WIoU assigns different weight gradients to training data of varying qualities, preventing the model from excessively focusing on challenging or easily identifiable examples, thus improving the model's stability.

Currently, the yarn cone dataset has fewer camera viewpoints and a relatively limited range of yarn cone and yarn warehouse poses, leading to challenges in detection accuracy under large tilt angles. Furthermore, the dataset includes a limited variety of yarn cone classifications. In future work, we plan to supplement the dataset with images capturing yarn cones and yarn warehouses from various angles and poses, enhancing yarn cone color classification, and introducing residue classification to further refine the yarn cone detection algorithm serving the yarn warehouse-type automatic winding machine.

## Data Availability

The datasets generated and/or analysed during the current study are not publicly available due [the sensitive commercial operations information contained within the dataset] but are available from the corresponding author on reasonable request.
